# Effect of arm position, presence of medical devices, and off-centering during acquisition of scout image on automatic tube voltage selection and current modulation in pediatric chest CT

**DOI:** 10.1371/journal.pone.0195807

**Published:** 2018-04-17

**Authors:** Young Jin Ryu, Young Hun Choi, Jung-Eun Cheon, Ji Eun Park, Woo Sun Kim, In-One Kim

**Affiliations:** 1 Department of Radiology, Seoul National University Hospital, Seoul, Korea; 2 Department of Radiology, Seoul National University College of Medicine, Seoul, Korea; 3 Institute of Radiation Medicine, Seoul National University Medical Research Center, Seoul, Korea; 4 Department of Radiology, Kyung Hee University Hospital, Graduate School, Kyung Hee University, Seoul, Korea; Medical University of Graz, AUSTRIA

## Abstract

**Purpose:**

To evaluate the patients’ morphologic factors affecting radiation dose in pediatric chest CT.

**Materials and methods:**

From November 2013 to May 2015, 315 pediatric chest CT scans were obtained using a CT scanner, and classified into 5 groups according to the patients’ age. For each age group, the chest CT scans were divided into two subgroups. A cut-off value used was the 75th percentile of size-specific dose estimates (SSDE), age-specific diagnostic reference level (DRL): less than the 75th percentile of SSDE (Group A, n = 238) and greater than the 75th percentile of SSDE (Group B, n = 77). All CT scans were performed with the same protocol using automatic tube voltage selection and current modulation techniques. The morphologic factors of the patients including body mass index (BMI), arm angles, presence of medical devices in the scan field, and degree of off-centering within the CT gantry were compared between groups A and B.

**Results:**

Group B showed narrower arm angles on scout and coronal reformatted images, higher frequency of the presence of devices and higher BMI than group A (*P* < 0.001, *P* < 0.001; *P* = 0.018, and *P* < 0.001, respectively). In multivariate analysis, narrower arm angles, the presence of devices on the scout images and higher BMI were independently associated with higher SSDE (*P* = 0.001, *P* = 0.037, and *P* < 0.001, respectively).

**Conclusions:**

During acquisition of the scout images, arms-down position and the presence of medical devices were associated with a high radiation dose above age-specific DRLs in pediatric chest CT, regardless of repositioning before the actual scanning. In addition, off-centering had no clinical impact on radiation dose in the routine practice.

## Introduction

Radiation exposure in children has become a great concern as CT images hold an important position in diagnostic algorithm and monitoring of therapeutic responses [1–3]. An important reason for radiologists to consider reducing radiation dose specifically in pediatric CT, is that children are at a greater risk of radiation-induced cancer development due to longer expected lifespan and higher radiosensitivity compared to adults [4, 5]. Thus, a number of dose-saving strategies and guidelines to optimize pediatric CT examinations have been developed [6, 7].

Recently, a method combining an automatic tube voltage selection (ATVS) technique and automatic tube current modulation (ATCM) technique was introduced to reduce radiation dose while maintaining image quality [8–11]. With this method, the optimal tube voltage is automatically selected for each patient, and the tube current is modulated in real-time manner. Even though the level of tube voltage and tube current are largely determined by the body size and morphology in these techniques, several ancillary factors, such as presence of devices, patient off-centering, or arm positioning, have been reported to be important for tube current modulation and voltage selection in previous studies [12–17].

However, to our knowledge, the complex influences of these morphologic factors on radiation doses have not been comprehensively analyzed in children [18]. Therefore, the purpose of this study was to evaluate the patients’ morphologic factors affecting radiation dose in pediatric chest CT.

## Materials and methods

This retrospective study was approved by the Seoul National University Hospital Institutional Review Board and the requirement for informed consent was waived.

### Patients

**Fig 1:**
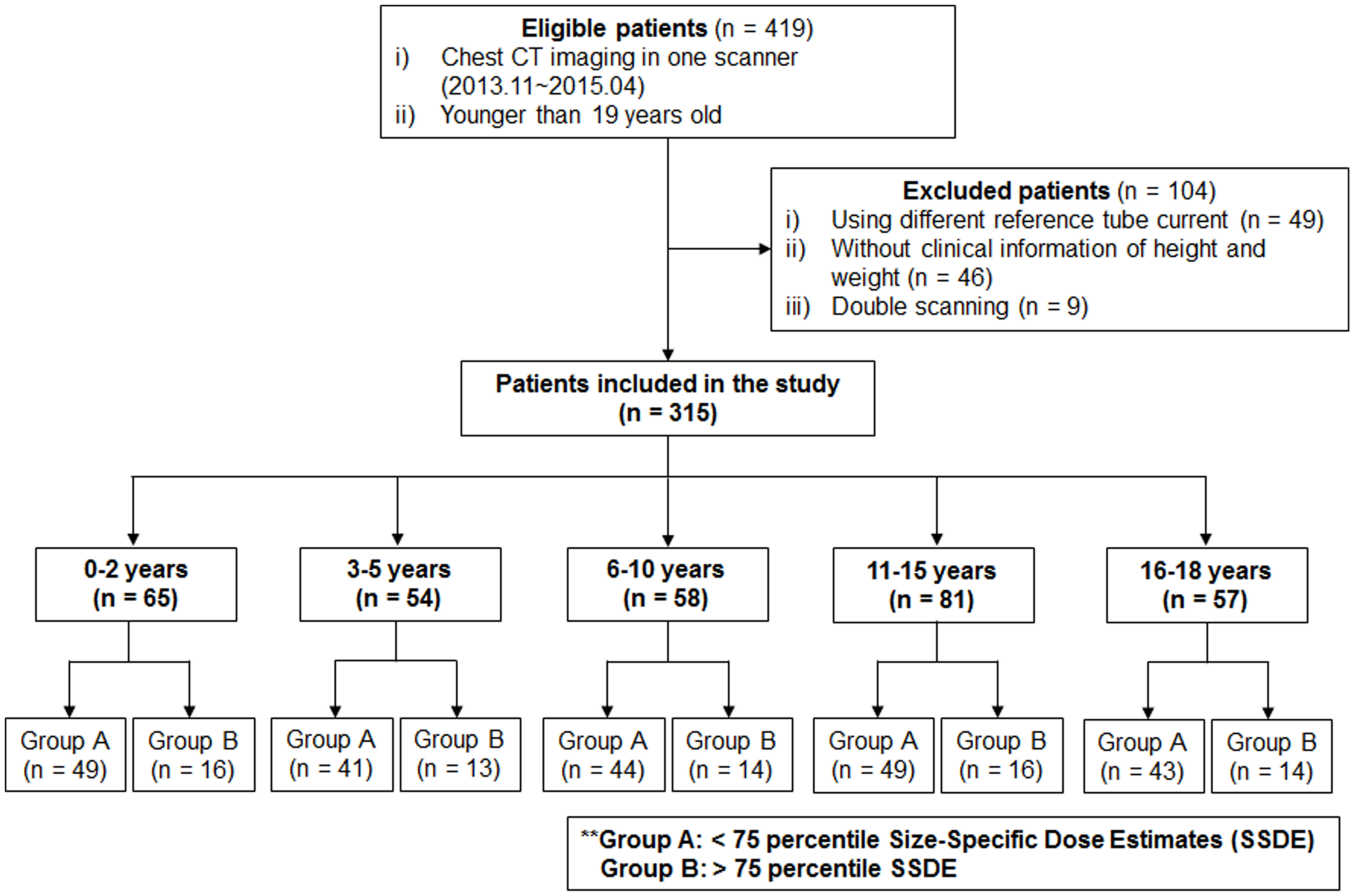
Flow diagram of the study population.

From November 2013 to May 2015, 419 pediatric chest CT examinations were performed on a CT scanner. One hundred and four CT scans were excluded due to the following causes: 1) CT scanning was performed using a non-reference tube current setting (n = 49); 2) patients without clinical information of height and weight (n = 46); and 3) double scanning was performed because of inadequate scanning at the initial exam (n = 9). A total of 315 CT examinations were included in this study. The patients were classified into 5 groups according to age (0–2 years, n = 65; 3–5 years, n = 54; 6–10 years, n = 58; 11–15 years, n = 81; 16–18 years, n = 57). The age groups were set as closely as possible to multiple nationwide surveys on the subject of diagnostic reference level (DRL) [19–23]. For each age group, chest CT scans were divided into two subgroups: Group A, less than the 75th percentile of the size-specific dose estimates (SSDE) for each age group, n = 238; and Group B, greater than the 75th percentile of the SSDE for each age group, n = 77, considering that the 75th percentile of a radiation dose distribution is used as a DRL [24]. A flow diagram of the study population is presented in Fig 1.

The dose-length product (DLP) and volume CT dose index (CTDI_vol32_) values were obtained from the dose reports and each SSDE was calculated according to the equation:

SSDE(mGy)=conversionfactor×CTDIvol(mGy).

A conversion factor was selected in each patient as a function of the sum of the lateral and AP dimensions measured on the CT image at the level of the left inferior pulmonary vein draining into the left atrium [25].

### CT technique

All CT scans were performed with a 128-section scanner (SOMATOM Definition Flash, Siemens Healthcare, Forchheim, Germany) using the same protocol according to an automatic tube voltage selection technique (Care kV) and an automated tube current modulation technique (CARE Dose4D) (reference tube voltage of 120 kV and reference tube current-time product of 100 mAs). One of the three different sets of tube voltage and tube current was selected for one CT scan: 120 kV 100 mAs, 100 kV 130 mAs, and 80 kV 303 mAs. A mid-level setting for the ATVS weighting (slider position 7 of 12) designed for soft-tissue examination was selected in our study to keep the contrast-to-noise ratio (CNR) balanced. All scout images were obtained using 80 kVp in an anteroposterior projection. All single phase contrast-enhanced chest CT scans were performed using the following parameters: detector configuration, 128 × 0.6 mm; gantry rotation time, 0.28 s; 180 x 180 mm field-of-view with an adjustment for each patient, 120 x 120 mm– 384 x 384 mm; resolution, 512 x 512; slice thickness, 3 mm; and reconstruction interval, 2 mm. Axial, coronal, and sagittal images were reconstructed from the raw volumetric data using an image space iterative reconstruction (ISIR) kernel (I30f) for all patients. Breathing instructions were provided only if the patient could hold his or her breath; if not, CT was performed with free breathing.

### Data analysis

Effective tube current and tube voltage were compared between groups A and B according to age. Sex ratio, age, weight, height, anteroposterior (AP) body diameter, and lateral diameter were compared between the two groups in each age group. In addition, body mass index (BMI), arm positioning and angles on the scout coronal images and coronal reformatted images, presence of medical devices in the scan field, and degree of off-centering within the CT gantry in vertical and lateral directions were also compared between groups A and B.

The height and weight of each patient were obtained from medical records and BMI was calculated. Maximum AP and lateral diameters were measured manually at the level of the left inferior pulmonary vein draining into the left atrium.

The angle of the arm was defined as the difference between the long axis of the humerus and the long axis of the torso, and quantified manually on both scout coronal images and coronal reformatted images. The averaged angles of the right and left arms were used for analysis. If arm angle on either side was less than 90 degrees, it was considered as an arms-down position. The arm angle measurements were performed by a pediatric radiologist (Y.J.R., with 6 years of experience). All identifying data were fully anonymized before the assessment.

Degree of off-centering was defined as the distance between the center of data collection and center of reconstruction target. Here we assumed that the center of reconstruction target would be the isocenter of the gantry and calculated by the equation in vertical and lateral directions, respectively:

$$\begin{array}{ll} \left(Off-centering\;in\;vertical\;direction\right) & =\left(Distance\;between\;centers\;of\;data\;collection\;and\;reconstruction\;target\;in\;y-axis\right)=y^{\prime }-y;\\ \left(Off-centering\;in\;lateral\;direction\right) & =\left(Distance\;between\;centers\;of\;data\;collection\;and\;reconstruction\;target\;in\;x-axis\right)=\;\sqrt{{\left(x^{\prime }-x\right)}^{2}} \end{array}$$

$$\left(x,y,coordinates\;of\;the\;center\;of\;gantery;x^{\prime },y^{\prime },coordinates\;of\;the\;center\;of\;reconstructed\;image\;;mm\right)$$

If the patients had a metallic device (screw, prosthesis, mechanical valve, ECG lead), endotracheal tube, bag valve mask or percutaneous drainage catheter in the scan field, they were considered as positive medical device cases. Plastic and thin (thickness < 1cm) devices such as a Levin tube, central line, or Hickmann catheter were not taken into account.

### Statistical analysis

An unpaired Student’s *t*-test was used to compare the continuous values such as age, tube current, weight, height, BMI, AP diameter, lateral diameter, angles of arm on the scout / reformatted coronal images, and degree of off-centering in vertical and lateral directions between groups A and B. The categorical scores, including sex ratio, position of arm (up or down), and presence of devices were compared between the two groups using a chi-square test or Fisher’s exact test. For an age group analysis, the Mann-Whitney U test was used to determine continuous scores between groups A and B due to a small number of subjects. To determine the strength of the association found between higher SSDE and variables, we performed forward stepwise logistic regression analysis with group B as the dependent outcome. Variables that were highly cross-correlated with BMI were not entered in the model to determine the best model predictive of higher SSDE. All statistical analyses were performed using SPSS (version 22.0, SPSS Inc., Chicago, IL, USA). Results with *P*-values < 0.05 were considered significant.

## Results

### Radiation dose

**Fig 2:**
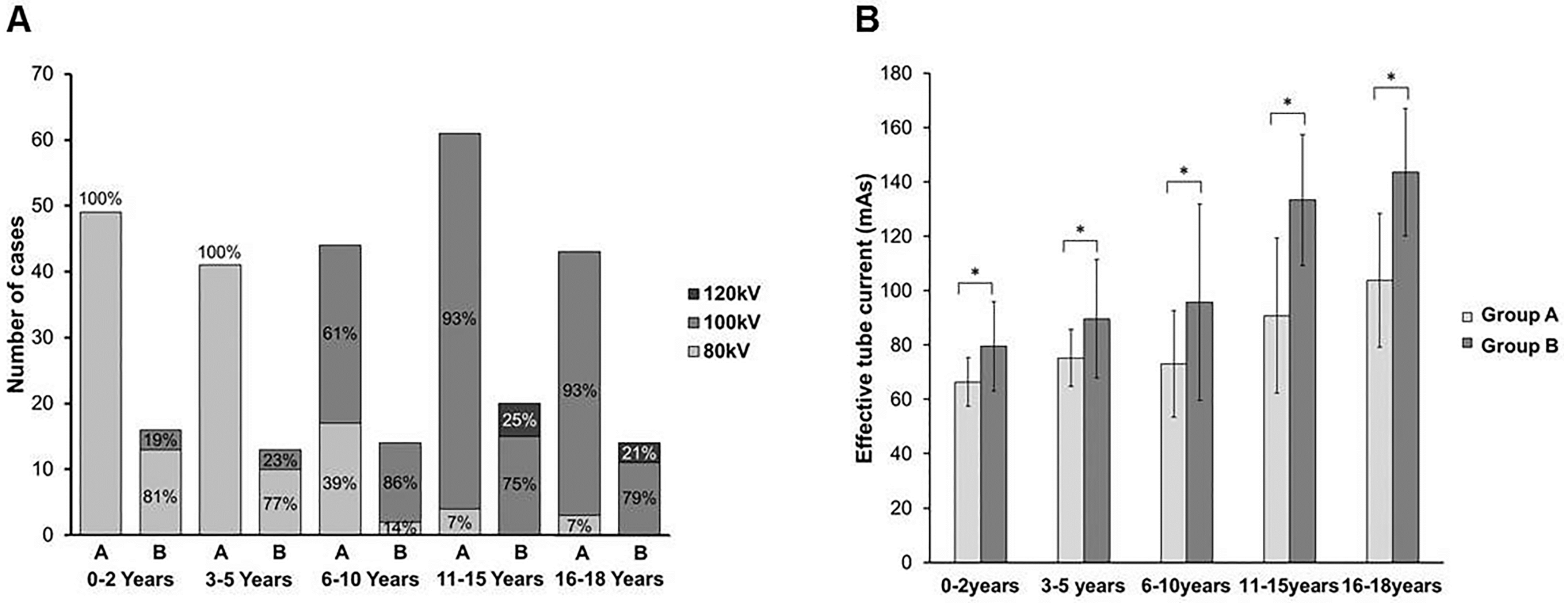
Distribution of selected tube voltages (A) and mean effective tube currents (B) in patient groups A and B in respective age groups. Asterisks denote significant differences between groups A and B (*p* < 0.05, unpaired t test). Error bars represent 1 SD above and below mean.

The distribution of radiation doses, including SSDE, CTDI_vol32_, and DLP, according to age groups is summarized in Table 1. With respect to SSDE, group B radiation exposure was 35–55% higher than that of group A. There was a larger proportion of higher tube voltage in group B than in group A in all age groups (Fig 2A). In addition, effective tube currents for chest CT in group B were significantly higher than those in group A (Fig 2B).

**Table 1:** Summary of SSDE, CTDI_vol32_, and DLP.

Age group	SSDE (mGy)		CTDI_vol32_ (mGy)		DLP (mGy·cm)	
	Mean ± SD	Range	Mean ± SD	Range	Mean ± SD	Range
**0–2 years**						
Total (n = 65)	2.5 ± 0.5	1.4–4.3	1.1 ± 0.2	0.5–1.9	23 ± 6	9–39
Group A (n = 49)	2.3 ± 0.2	1.4–2.7	1.1 ± 0.1	0.5–1.3	21 ± 6	9–39
Group B (n = 16)	3.1 ± 0.5	2.7–4.3	1.4 ± 0.2	1.1–1.9	28 ± 5	17–35
**3–5 years**						
Total (n = 54)	2.6 ± 0.5	1.6–4.0	1.3 ± 0.3	0.8–2.0	30 ± 7	16–45
Group A (n = 41)	2.4 ± 0.3	1.6–2.8	1.2 ± 0.2	0.8–1.5	28 ± 6	16–39
Group B (n = 13)	3.3 ± 0.3	2.8–4.0	1.6 ± 0.2	1.4–2.0	38 ± 4	33–45
**6–10 years**						
Total (n = 58)	3.7 ± 0.8	1.5–5.4	2 ± 0.5	0.8–3.4	55 ± 15	25–90
Group A (n = 44)	3.4 ± 0.6	1.5–4.2	1.8 ± 0.3	0.8–2.3	50 ± 12	25–80
Group B (n = 14)	4.7 ± 0.4	4.2–5.4	2.8 ± 0.3	2.1–3.4	71 ± 13	50–90
**11–15 years**						
Total (n = 81)	5.2 ± 1.5	3.5–12.3	3.4 ± 1.4	2.1–10.1	115 ± 50	51–313
Group A (n = 61)	4.6 ± 0.6	3.5–5.7	2.8 ± 0.5	2.1–4.2	94 ± 24	51–159
Group B (n = 20)	7.1 ± 1.8	5.7–12.3	5.2 ± 1.7	3.6–10.1	178 ± 58	110–313
**16–18 years**						
Total (n = 57)	5.6 ± 1.5	3.2–11.9	3.8 ± 1.4	1.9–9.2	135 ± 49	58–346
Group A (n = 43)	4.9 ± 0.8	3.2–6.2	3.3 ± 0.7	1.9–4.7	117 ± 26	58–171
Group B (n = 14)	7.6 ± 1.6	6.3–11.9	5.4 ± 1.7	4.0–9.2	193 ± 60	127–346

Note.–SSDE = Size-specific dose estimates, CTDI_vol_ = Volume CT dose index, DLP = Dose-length product

### Age group analysis

Table 2 shows demographics and morphologic factors of group A and group B according to age groups. In all five age groups, there was no statistically significant difference between group A and group B with respect to sex ratio and age. Body weight and lateral diameter were higher in group B than in group A in patients aged 6 years or older. Height and AP diameter were significantly higher in group B than in group A in patients aged 6–15 years. In patients who were 0–2 or 3–5 years old, there was no statistically significant difference between group A and group B with respect to any of the demographic and morphologic characteristics.

**Table 2:** Demographics and morphologic factors of patients.

	Sex (M/F)	Age (years)	Weight (kg)	Height (cm)	AP diameter (cm)	Lat. diameter (cm)
**0-2years**						
Group A	26/23	1.7 ± 0.7	10.9 ± 2.8	81 ± 10	11.6 ± 0.8	17.3 ± 1.9
Group B	11/5	1.8 ± 0.7	11.1 ± 2.3	81 ± 9	11.9 ± 0.7	17.3 ± 1.5
*p*-value*	0.271^†^	0.67	0.67	0.879	0.217	0.843
**3–5 years**						
Group A	19/22	4.5 ± 0.7	15.3 ±2.9	102 ± 8	13.2 ± 0.9	20.1 ± 1.8
Group B	10/3	4.3 ± 0.8	17.1 ± 4.5	103 ± 10	14.1 ± 2.0	20.5 ± 2.9
*p*-value*	0.054^†^	0.511	0.479	0.863	0.172	0.297
**6-10years**						
Group A	27/17	8.4 ± 1.3	23.9 ± 4.2	125 ± 8	15.2 ± 0.9	23.1 ± 1.5
Group B	7/7	8.8 ± 1.4	30.4 ± 6.2	131 ± 9	16.5 ± 1.6	25.8 ± 2.5
*p*-value*	0.452^†^	0.326	<0.001	0.039	0.006	<0.001
**11-15years**						
Group A	32/29	13.3 ± 1.2	42.2 ± 9.9	157 ± 8	17.7 ± 1.8	27.9 ± 3.0
Group B	13/7	13.8 ± 1.3	61.1 ± 12.4	165 ± 10	21.1 ± 2.2	33.0 ± 3.5
*p*-value*	0.327^†^	0.119	<0.001	0.004	<0.001	<0.001
**16-18years**						
Group A	33/10	16.9 ± 0.5	50.2 ± 9.6	167 ± 9	18.9 ± 1.7	30.5 ± 2.6
Group B	9/5	16.8 ± 0.6	59.2 ± 10.5	166 ± 12	19.6 ± 3.0	33.9 ± 2.6
*p*-value*	0.486^‡^	0.711	0.006	0.963	0.553	<0.001

Note.–Values are the means ± standard deviation. AP = anteroposterior, Lat. = Lateral

* Significant difference between two groups (*P* < .05). The difference between two groups was evaluated using the Mann-Whitney U test in cases of continuous variables and using a chi-square test(^†^) or Fisher’s exact test(^‡^) in cases of categorical variables.

### Factors affecting radiation dose

A total of 34 (10.8%) examinations were performed with arms down during scout acquisition. With the exception of two cases, all actual CT images were acquired with arms above the shoulders by changing arm position just before actual CT scanning.

Group B showed significantly higher BMI, narrower arm angles on the scout coronal images and coronal reformatted images, higher frequency of the arms-down position on the scout images and the presence of medical devices in the scanning field (Table 3). However, significant differences were not observed between groups A and B with regard to off-centering in vertical and lateral directions and frequency of the arms-down position on actual scanning (Table 3).

**Table 3:** Comparison of morphologic factors affecting radiation doses between the two groups.

Variable	Group A (n = 238)	Group B (n = 77)	*p*-value*
BMI (kg/m^2^)	16.3 ± 2.8	19.1 ± 3.7	< 0.001
Position of arm on scout image (up / down) on scout coronal image	226 / 12	55 / 22	< 0.001^†^
Angle of arm on scout coronal image (°)	140 ± 29	111 ± 48	< 0.001
Position of arm on scout image (up / down) on coronal reformatted image	237 / 1	76 / 1	0.430^‡^
Angle of arm on coronal reformatted image (°)	146 ± 13	137 ± 15	< 0.001
Device (− / +)	224 / 14	66 / 11	0.018^†^
Off-centering in vertical direction (mm)	-1.8 ± 7.8	0.2 ± 10.7	0.076
Off-centering in lateral direction (mm)	7.3 ± 5.5	8.1 ± 6.5	0.273

Note.–Values are the means ± standard deviation. BMI = body mass index

* Significant difference between two groups (*P<* 0.05). The difference between two groups was evaluated using an unpaired student’s t-test in cases of continuous variables and using a chi-square test(^†^) or Fisher’s exact test(^‡^) in cases of categorical variables.

In addition, group B had significantly higher weights and longer AP/lateral diameters compared to group A (weight, 37.4 ± 23.0 kg vs. 29.2 ± 16.7 kg, *P* = 0.005; AP diameter, 16.9 ± 4.1 cm vs. 15.4 ± 3.0 cm, *P* = 0.004; lateral diameter, 26.5 ± 7.2 cm vs. 24.0 ± 5.4 cm, *P* = 0.006). There were no significant differences between the two groups in sex ratio, age, and height (Group A vs. group B: male/female, 137/101 vs. 50/27, *P* = 0.252; age, 9.1 ± 5.6 years vs. 9.3 ± 5.8 years, *P* = 0.78; height, 128 ± 34 cm vs. 131 ± 36 cm, *P* = 0.505).

**Fig 3:**
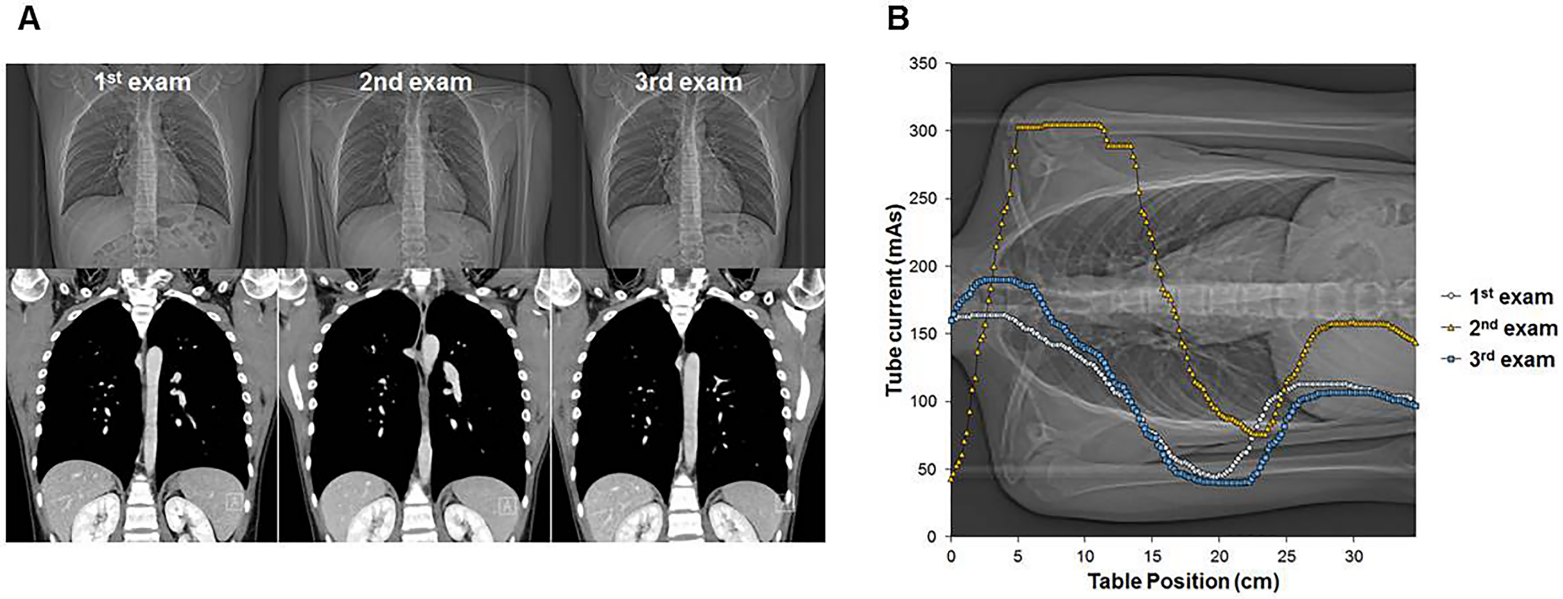
Chest CT images in a 16-year-old boy with Hodgkin’s lymphoma. (**A)** A series of three chest CT scans obtained at an interval of 3 months are shown: scout images of upper column and coronal reformatted images of lower column. At 1^st^ and 3^rd^ CT examinations, scout images were taken while holding arms up. At 2^nd^ CT examination, scout image was acquired during an arms-down position. All three real CT images were acquired with arms-up positions (shown in coronal reformatted images). (**B**) The graph shows the average effective tube current/slice for each CT scan. SSDE and effective tube current of the 2^nd^ exam (group B, 7.59 mGy, 172 mAs, respectively) were higher than those of the 1^st^ and 3^rd^ exams (1^st^ exam, group A, 4.81 mGy, 111 mAs; 3^rd^ exam, group A, 4.73 mGy, 109 mAs, respectively). The highest tube current/rotation of the 2^nd^ CT scan was observed at the level of the proximal humerus. There were no differences among three acquisitions with respect to BMI, height, weight, AP/lateral diameters and tube voltage (100 kV).

**Fig 4:**
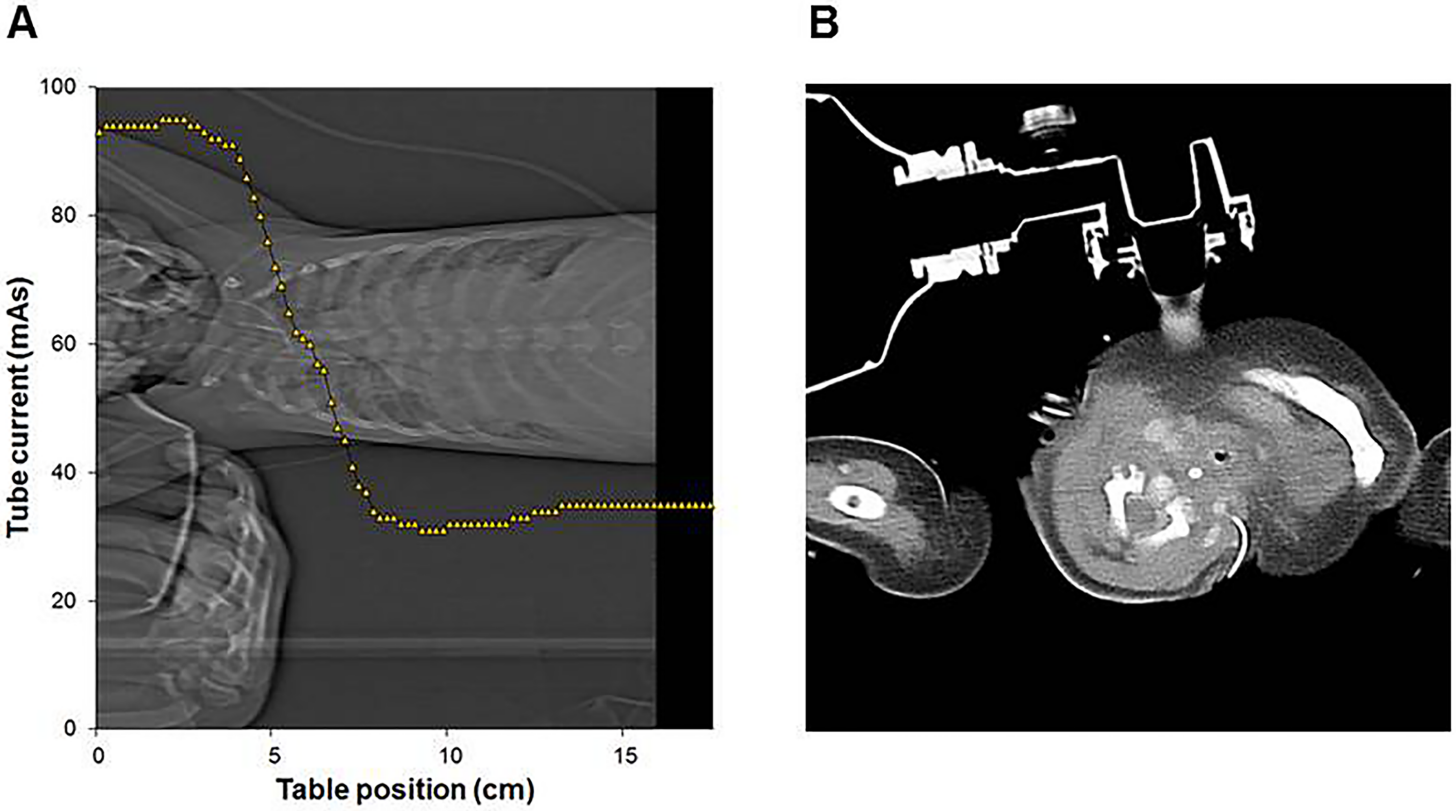
Chest CT images in a 10-month-old boy with septic shock. On the scanogram (A), the bag valve mask and an adult hand were included in the FOV of the scan, and a corresponding CT image (B) shows a bag valve mask. The graph (A) shows the average effective tube current/slice in the CT scan. The highest effective tube current was observed at the level of the neck and upper thorax where the unintended objects were located. In addition, 100 kV of tube voltage was used for the scan, while most scans were obtained with 80kV in the same age group (0–2 years old). The SSDE was 4.32 mGy and this patient was classified into group B.

BMI, arm angles on the scout coronal images, presence of medical devices in the scanning field, and degree of off-centering in the lateral direction were included in the multivariate analysis. Narrower arm angles and presence of devices on the scout images as well as higher BMI were independently associated with higher SSDE (Table 4, Figs 3 and 4).

**Table 4:** Multivariate logistic regression analysis of factors affecting radiation doses.

Variable	Adjusted Odds ratio	*p*-value*
BMI	1.318 (1.203, 1.444)	< 0.001
Angle of arm on scout coronal image	0.981 (0.974, 0.989)	< 0.001
Presence of device	3.335 (1.295, 8.587)	0.013
Off-centering in vertical direction (mm)	NA	0.330

Note.–The data in parentheses are 95% confidence intervals. BMI = body mass index, NA = not applicable

* A *P*-value less than 0.05 indicates statistical significance.

## Discussion

In our study, narrow arm angles and presence of medical devices on the scout images as well as the high BMI were independently associated with high SSDE, while off-centering was not associated with high SSDE. Although arms were repositioned before the actual scanning in most patients, arm position on the scout images influenced the radiation dose. Thus, we believe that radiation dose may be effectively reduced by simple measures such as repositioning the arms from a downward to upward position and taking away medical devices from the scan field before taking the scout image.

As expected, those with higher BMI had higher SSDE because ATCM and ATVS basically operate according to body size and morphology, and the purpose of ATCM and ATVS is to maintain image quality regardless of body size [26].

It is well-known that arm positioning is an important factor in chest CT for determining radiation dose. For the ATVS technique, overall patient size and patient attenuation measured from the scout image primarily determine the tube voltage. Furthermore, the z-axis ATCM implemented for estimating the tube current along the z-axis was influenced mostly by the patient size and attenuation obtained from the scout image [9, 27]. Therefore, a high radiation dose was observed in patients with the scout image taken in an arm-down position. The arm-down position increased the overall patient size, which led to selection of a higher tube voltage and tube current than actually required values. The previous studies also showed that positioning of the arms alongside the torso led to an increased radiation dose in trauma CT for thoracoabdominal region, and the authors advocated raising arms above the shoulder level to achieve a lower radiation dose and better image quality [17, 28].

In this study many patients changed arm positioning from alongside the trunk for the acquisition of the scout image to above the shoulder after taking the scout image. This acquisition sequence was taking place since many patients at our institution were asked to undergo a brain or neck CT scan simultaneously with chest CT. In this situation, radiology technicians randomly selected an arm position at the time of the scout image acquisition. After that, chest CT images were obtained with the arms up, while brain or neck CT was performed with an arms-down position to obtain better quality image. In this study, we found that an arms-down position during the scout image would increase radiation dose even after the arm position is corrected from downward to upward before the actual tube rotation, which is consistent with the results of a previous case report [29]. Therefore, the manufacturer does not recommend changes in arm position between a topogram scan and a corresponding CT scan for proper functioning of CARE Dose4D [30]. If two different body parts are scanned consecutively with different arm positions in one patient, acquiring two topograms would be better for proper functioning of CARE Dose4D.

When it comes to the presence of medical devices in the scanning field, it can be concluded that the presence of a large medical device, such as a bag valve mask would increase both the patient’s attenuation and apparent size, resulting in the increased tube current and voltage selection with the use of ATCM and ATVS.

Interestingly, off-centering of the patient was not associated with the higher SSDE in this study, which was not in accordance with the results of previous studies [14–16]. A study by Kaasalainen et al. reported that radiation doses increased by 21% and 12% with 6-cm vertical off-centering for pediatric 5-year-old and newborn phantoms, respectively, because of a magnification effect of the scout image [14]. However, in clinical practice at our institution, off-centering, especially vertical off-centering, was not significant and was in the sub-centimeter range. We think that off-centering is not significant in clinical practice because a laser-position marking system is routinely available in a modern CT machine.

Radiation dose used in our study was lower than those in other studies performed for the purpose of national survey. The SSDE in the study investigating pediatric CT radiation doses in South Korea were 1.6–2 times higher than the SSDE in this study; even though approximately 40% of CT scans reported were multiphase scans, radiation dose in that study was higher than in our study [19]. Moreover, the authors stated that radiation doses in their study were generally lower than in the UK survey [20].

There were some limitations in our study. First, the attenuation of lung parenchyma may be a confounding variable and we did not consider its effect on tube current and tube voltage. In patients with severe parenchymal disease, such as pneumonia, there is a high probability that a bag valve mask or adult hands are present in the scanned field. Second, quantitative or qualitative analysis of image quality was not performed. However, several studies investigating the effect of arm position on radiation dose and image quality showed the degradation of image quality and increase of noise in the upper abdomen, but not in the thorax, with positioning of the arms alongside the torso [17, 28, 31]. Thus, we assumed that all CT examinations showed similar and acceptable image quality. Third, the definition of medical devices was rather subjective because the medical devices had a wide range of sizes and attenuation.

## Conclusion

During acquisition of the scout images, arms-down position and the presence of medical devices were associated with a high radiation dose above age-specific DRLs in pediatric chest CT, regardless of repositioning before the actual scanning. In addition, off-centering had no clinical impact on radiation dose in the routine practice.

## Supporting information

S1 File Patients information and CT radiation dose. (XLSX)

## Abbreviations

AP: Anteroposterior
ATCM: Automatic tube current modulation
ATVS: Automatic tube voltage selection
BMI: Body mass index
CTDI_vol_: Volume CT dose index
DLP: Dose-length product
DRL: Diagnostic reference level
SSDE: Size-specific dose estimates

## References

[1] Dougeni E, Faulkner K, Panayiotakis G. A review of patient dose and optimisation methods in adult and paediatric CT scanning. European journal of radiology. 2012;81(4):e665–e83. doi: 10.1016/j.ejrad.2011.05.025 21684099 10.1016/j.ejrad.2011.05.025

[2] Pearce MS. Patterns in paediatric CT use: an international and epidemiological perspective. Journal of medical imaging and radiation oncology. 2011;55(2):107–9. doi: 10.1111/j.1754-9485.2011.02240.x 21501396 10.1111/j.1754-9485.2011.02240.x

[3] Brenner DJ, Hall EJ. Computed Tomography—An Increasing Source of Radiation Exposure. N Engl J Med. 2007;357:2277–84. doi: 10.1056/NEJMra072149 18046031 10.1056/NEJMra072149

[4] Smith-Bindman R, Lipson J, Marcus R, Kim K-P, Mahesh M, Gould R, et al Radiation dose associated with common computed tomography examinations and the associated lifetime attributable risk of cancer. Archives of internal medicine. 2009;169(22):2078–86. doi: 10.1001/archinternmed.2009.427 20008690 10.1001/archinternmed.2009.427 PMC4635397

[5] Pearce MS, Salotti JA, Little MP, McHugh K, Lee C, Kim KP, et al Radiation exposure from CT scans in childhood and subsequent risk of leukaemia and brain tumours: a retrospective cohort study. The Lancet. 2012;380(9840):499–505. 10.1016/S0140-6736(12)60815-0 PMC3418594 22681860

[6] Strauss KJ, Goske MJ, Kaste SC, Bulas D, Frush DP, Butler P, et al Image gently: ten steps you can take to optimize image quality and lower CT dose for pediatric patients. American Journal of Roentgenology. 2010;194(4):868–73. doi: 10.2214/AJR.09.4091 20308484 10.2214/AJR.09.4091

[7] Goo HW. CT radiation dose optimization and estimation: an update for radiologists. Korean journal of radiology. 2012;13(1):1–11. doi: 10.3348/kjr.2012.13.1.1 22247630 10.3348/kjr.2012.13.1.1 PMC3253393

[8] Lee KH, Lee JM, Moon SK, Baek JH, Park JH, Flohr TG, et al Attenuation-based automatic tube voltage selection and tube current modulation for dose reduction at contrast-enhanced liver CT. Radiology. 2012;265(2):437–47. 23012467 10.1148/radiol.12112434

[9] Siegel MJ, Hildebolt C, Bradley D. Effects of automated kilovoltage selection technology on contrast-enhanced pediatric CT and CT angiography. Radiology. 2013;268(2):538–47. 23564712 10.1148/radiol.13122438

[10] Hough DM, Fletcher JG, Grant KL, Fidler JL, Yu L, Geske JR, et al Lowering kilovoltage to reduce radiation dose in contrast-enhanced abdominal CT: initial assessment of a prototype automated kilovoltage selection tool. American Journal of Roentgenology. 2012;199(5):1070–7. doi: 10.2214/AJR.12.8637 23096181 10.2214/AJR.12.8637

[11] Winklehner A, Goetti R, Baumueller S, Karlo C, Schmidt B, Raupach R, et al Automated attenuation-based tube potential selection for thoracoabdominal computed tomography angiography: improved dose effectiveness. Investigative radiology. 2011;46(12):767–73. 21730872 10.1097/RLI.0b013e3182266448

[12] Geleijns J, Wang J, McCollough C. The use of breast shielding for dose reduction in pediatric CT: arguments against the proposition. Pediatric radiology. 2010;40(11):1744–7. doi: 10.1007/s00247-010-1808-2 20730422 10.1007/s00247-010-1808-2

[13] Coursey C, Frush DP, Yoshizumi T, Toncheva G, Nguyen G, Greenberg SB. Pediatric chest MDCT using tube current modulation: effect on radiation dose with breast shielding. American Journal of Roentgenology. 2008;190(1):W54–W61. doi: 10.2214/AJR.07.2017 18094273 10.2214/AJR.07.2017

[14] Kaasalainen T, Palmu K, Reijonen V, Kortesniemi M. Effect of patient centering on patient dose and image noise in chest CT. American Journal of Roentgenology. 2014;203(1):123–30. doi: 10.2214/AJR.13.12028 24951205 10.2214/AJR.13.12028

[15] Li J, Udayasankar UK, Toth TL, Seamans J, Small WC, Kalra MK. Automatic patient centering for MDCT: effect on radiation dose. American Journal of Roentgenology. 2007;188(2):547–52. doi: 10.2214/AJR.06.0370 17242267 10.2214/AJR.06.0370

[16] Matsubara K, Koshida K, Ichikawa K, Suzuki M, Takata T, Yamamoto T, et al Misoperation of CT automatic tube current modulation systems with inappropriate patient centering: phantom studies. American Journal of Roentgenology. 2009;192(4):862–5. doi: 10.2214/AJR.08.1472 19304687 10.2214/AJR.08.1472

[17] Brink M, de Lange F, Oostveen LJ, Dekker HM, Kool DR, Deunk J, et al Arm Raising at Exposure-controlled Multidetector Trauma CT of Thoracoabdominal Region: Higher Image Quality, Lower Radiation Dose 1. Radiology. 2008;249(2):661–70. 18936319 10.1148/radiol.2492080169

[18] Cody DD. Management of auto exposure control during pediatric computed tomography. Pediatric radiology. 2014;44(3):427–30. 25304700 10.1007/s00247-014-3140-8

[19] Hwang J-Y, Do K-H, Yang DH, Cho YA, Yoon H-K, Lee JS, et al A Survey of Pediatric CT Protocols and Radiation Doses in South Korean Hospitals to Optimize the Radiation Dose for Pediatric CT Scanning. Medicine. 2015;94(50):e2146 26683922 10.1097/MD.0000000000002146 PMC5058894

[20] Shrimpton P, Hillier M, Lewis M, Dunn M. National survey of doses from CT in the UK: 2003. The British journal of radiology. 2006;79(948):968–80. doi: 10.1259/bjr/93277434 17213302 10.1259/bjr/93277434

[21] Takei Y, Miyazaki O, Matsubara K, Shimada Y, Muramatsu Y, Akahane K, et al Nationwide survey of radiation exposure during pediatric computed tomography examinations and proposal of age-based diagnostic reference levels for Japan. Pediatric radiology. 2016;46(2):280–5. doi: 10.1007/s00247-015-3474-x 26494635 10.1007/s00247-015-3474-x

[22] Granata C, Origgi D, Palorini F, Matranga D, Salerno S. Radiation dose from multidetector CT studies in children: results from the first Italian nationwide survey. Pediatric radiology. 2015;45(5):695–705. doi: 10.1007/s00247-014-3201-z 25380999 10.1007/s00247-014-3201-z

[23] Verdun FR, Gutierrez D, Vader JP, Aroua A, Alamo-Maestre LT, Bochud F, et al CT radiation dose in children: a survey to establish age-based diagnostic reference levels in Switzerland. European radiology. 2008;18(9):1980–6. doi: 10.1007/s00330-008-0963-4 18389242 10.1007/s00330-008-0963-4

[24] Strauss KJ, Goske MJ, Towbin AJ, Sengupta D, Callahan MJ, Darge K, et al Pediatric Chest CT Diagnostic Reference Ranges: Development and Application. Radiology. 2017:161530. 10.1148/radiol.2017161530 28212059

[25] Boone J, Strauss K, Cody D, McCollough C, McNitt-Gray M, Toth T. AAPM report No. 204: size-specific dose estimates (SSDE) in pediatric and adult body CT examinations. 2013.

[26] Kalra MK, Maher MM, Toth TL, Schmidt B, Westerman BL, Morgan HT, et al Techniques and applications of automatic tube current modulation for CT 1. Radiology. 2004;233(3):649–57. 15498896 10.1148/radiol.2333031150

[27] Lee CH, Goo JM, Ye HJ, Ye S-J, Park CM, Chun EJ, et al Radiation Dose Modulation Techniques in the Multidetector CT Era: From Basics to Practice 1. Radiographics. 2008;28(5):1451–9. 18794318 10.1148/rg.285075075

[28] Karlo C, Gnannt R, Frauenfelder T, Leschka S, Brüesch M, Wanner GA, et al Whole-body CT in polytrauma patients: effect of arm positioning on thoracic and abdominal image quality. Emergency radiology. 2011;18(4):285–93. doi: 10.1007/s10140-011-0948-5 21472460 10.1007/s10140-011-0948-5

[29] Kuo F, Plaza M, Saigal G. Inappropriate arm positioning during scout image acquisition resulting in increased radiation dose while performing a chest CT. Pediatric radiology. 2012;42(4):508–9. doi: 10.1007/s00247-011-2292-z 22322628 10.1007/s00247-011-2292-z

[30] Söderberg M. OVERVIEW, PRACTICAL TIPS AND POTENTIAL PITFALLS OF USING AUTOMATIC EXPOSURE CONTROL IN CT: SIEMENS CARE DOSE 4D. Radiation protection dosimetry. 2016;169(1–4):84–91. doi: 10.1093/rpd/ncv459 26567324 10.1093/rpd/ncv459

[31] Fanucci E, Fiaschetti V, Rotili A, Floris R, Simonetti G. Whole body 16-row multislice CT in emergency room: effects of different protocols on scanning time, image quality and radiation exposure. Emergency radiology. 2007;13(5):251–7. doi: 10.1007/s10140-006-0554-0 17180674 10.1007/s10140-006-0554-0

